# Ribozyme Assays to Quantify the Capping Efficiency of In Vitro-Transcribed mRNA

**DOI:** 10.3390/pharmaceutics14020328

**Published:** 2022-01-29

**Authors:** Irena Vlatkovic, János Ludwig, Gábor Boros, Gábor Tamás Szabó, Julia Reichert, Maximilian Buff, Markus Baiersdörfer, Jonas Reinholz, Azita Josefine Mahiny, Uğur Şahin, Katalin Karikó

**Affiliations:** 1 BioNTech SE, An der Goldgrube 12, 55131 Mainz, Germany; Gabor.Boros@biontech.de (G.B.); gabor.szabo@biontech.de (G.T.S.); julia.reichert@biontech.de (J.R.); Maximilian.Buff@biontech.de (M.B.); Markus.Baiersdoerfer@biontech.de (M.B.); jonas.reinholz@biontech.de (J.R.); Azita.Mahiny@biontech.de (A.J.M.); ugur.sahin@biontech.de (U.Ş.); katalin.kariko@biontech.de (K.K.); 2 Institute of Clinical Chemistry and Clinical Pharmacology, Rheinische Friedrich-Wilhelms-Universität Bonn, 53127 Bonn, Germany; janos.ludwig@uni-bonn.de

**Keywords:** mRNA capping efficiency, ribozyme, in vitro-transcribed (IVT) mRNA, cap, quality control

## Abstract

The presence of the cap structure on the 5′-end of in vitro-transcribed (IVT) mRNA determines its translation and stability, underpinning its use in therapeutics. Both enzymatic and co-transcriptional capping may lead to incomplete positioning of the cap on newly synthesized RNA molecules. IVT mRNAs are rapidly emerging as novel biologics, including recent vaccines against COVID-19 and vaccine candidates against other infectious diseases, as well as for cancer immunotherapies and protein replacement therapies. Quality control methods necessary for the preclinical and clinical stages of development of these therapeutics are under ongoing development. Here, we described a method to assess the presence of the cap structure of IVT mRNAs. We designed a set of ribozyme assays to specifically cleave IVT mRNAs at a unique position and release 5′-end capped or uncapped cleavage products up to 30 nt long. We purified these products using silica-based columns and visualized/quantified them using denaturing polyacrylamide gel electrophoresis (PAGE) or liquid chromatography and mass spectrometry (LC–MS). Using this technology, we determined the capping efficiencies of IVT mRNAs with different features, which include: Different cap structures, diverse 5′ untranslated regions, different nucleoside modifications, and diverse lengths. Taken together, the ribozyme cleavage assays we developed are fast and reliable for the analysis of capping efficiency for research and development purposes, as well as a general quality control for mRNA-based therapeutics.

## 1. Introduction

In vitro-transcribed (IVT) mRNA-based therapeutics are emerging as novel biologics with a variety of applications, including recent vaccines against coronavirus disease 2019 (COVID-19) caused by severe acute respiratory syndrome coronavirus 2 (SARS-CoV-2), and other infectious disease vaccine candidates, cancer immunotherapies, gene-editing treatments, and protein replacement therapies [1,2,3,4,5,6]. Ongoing preclinical and clinical studies using IVT mRNAs require innovative solutions for quality control of the IVT mRNA.

The presence of the cap structure at its 5’ terminus is one of the key features of IVT mRNA that increases its stability and translatability. Capped mRNAs are generally translated more efficiently compared to uncapped mRNAs [7,8,9]. After synthesis, enzymatic and co-transcriptional capping of IVT mRNA may be incomplete, leading to the presence of a variable number of uncapped molecules in the final IVT mRNA. Therefore, we developed assays that allow for fast and simple quantitative measurements of the capping efficiency. These assays can be used as a quality control for mRNA-based therapeutics.

Current approaches for determining the capping efficiency of IVT mRNA have limited applicability. In one method, IVT mRNA is transcribed from a short DNA template in the presence of [α-32P]GTP and a cap analog. Digestion of this generated IVT mRNA with RNase T2 has been shown to release a radioactive cap structure containing [32P]-labeled 3′-phosphate using anion exchange chromatography and subsequent measurements [10,11]. However, this approach is only applicable for RNAs < 50 nt long. Others have determined the efficiency of enzymatic capping of long mRNA transcribed in the presence of [γ-32P]GTP by measuring the reduction of mRNA radioactivity, as successful capping eliminates the labeled Gamma phosphate [12]. The major disadvantage of these methods is the use of radioactive material for mRNA production.

Another novel approach was developed whereby the capping efficiency of IVT mRNAs of different lengths (>1000 nt) was measured without the use of radioactivity. In this procedure, a complementary biotin-tagged oligonucleotide was annealed to the 5′-end of the IVT mRNA which was then cleaved by RNase H [13]. The cleaved 5′-end was purified using streptavidin-coated magnetic beads and then analyzed by liquid chromatography and mass spectrometry (LC–MS). Although this method avoids radiolabeling of the mRNA, the RNase H cleavage site was not unique since additional cleavage products of different lengths were also generated, making the method not fully reliable. Furthermore, analysis of the cleaved fragments requires LC–MS, which impacts the affordability of the assay. Recently, a biosensor detecting both the capping level and integrity of mRNA was developed [14]. However, the biosensor method only allows for the detection of variations in capping level in increments of at least 20%, making this method difficult to use as a precise analytical tool.

In the method described here, ribozymes (Rz) were designed to specifically cleave IVT mRNA at a unique position in close proximity to the 5′-end, releasing capped or uncapped short 5′ cleavage products in a range of 10–30 nt. The well-defined 5′ cleavage products cut off by the ribozyme from the capped mRNA differ by one nucleoside in length, specifically the cap structure itself, compared to the cleavage products cut from an uncapped RNA. RNAs from the cleavage reaction can be analyzed directly in denaturing polyacrylamide gel electrophoresis (PAGE) or further purified using a silica-based column and loading the resulting cleaned, short 5′ cleavage product RNAs onto the gel. The RNAs are electrophoresed under conditions in which the difference of the 5′ cleavage products is detectable. The stained and visualized 5′ cleavage products released from the capped or uncapped mRNAs are quantified via their gel band intensity, and the capping efficiency is assessed. In addition, the short purified 5′ cleavage products can be analyzed using LC–MS, allowing for an additional characterization, such as determination of methylation status or minor capped products.

## 2. Materials and Methods

### 2.1. Templates for In Vitro Transcription

Templates for in vitro transcription were generated by linearizing plasmids containing different coding sequences, flanked by sequences corresponding to the 5′ untranslated region (UTR) of human alpha globin (hAg) or 5′-leader of tobacco etch virus (TEV), a constant 3′UTR, and 100 nt long poly(A) tail [15,16]. The linearization was performed with the restriction enzymes EarI or BbsI (both from New England Biolabs, Ipswich, MA, USA).

### 2.2. In Vitro Transcription and Capping of RNA

RNA ranging in size from 100 nt to 9.4 kb was synthesized using the MEGAscript T7 transcription kit (Thermo Fisher Scientific, Invitrogen, Waltham, MA, USA). The reaction included UTP for generating standard IVT mRNA or N1-methylpseudouridine 5′-triphosphate (m1ΨTP) (TriLink) for nucleoside modified mRNA, in which 100% of uridine was substituted with m1Ψ. In a subset of RNAs, the sequences of the first three transcribed nucleotides were GCG, GGA, AGC, AGG or AGA. Cap analog, ARCA-G (TriLink, N-7003), beta-S-ARCA (D1, BioNTech SE) [17] or CleanCap^®^ Reagent AG (3′ OMe) (TriLink, N-7413) were added to the transcription reaction to generate mRNA with cap0 (A0), cap0 (D1) or cap1 (CC1), respectively. Vaccinia virus capping enzymes (New England Biolabs) were used according to the manufacturer’s instructions to enzymatically cap the synthesized mRNA and generate RNA with cap0 (E0) or cap1 (E1). RNA quality was tested using 1.4% agarose gel electrophoresis [18], and RNA concentration was measured using a NanoDrop spectrophotometer (Thermo Fisher Scientific).

### 2.3. Design of Ribozymes and RNase H Probe

Five hammerhead ribozymes were designed for this study (Table 1).

Rz1, Rz2, Rz3, and Rz4 cleave the hAg 5′UTR after GUC, GUA, GUC, and GUC triplets, respectively [19,20]. Rz3 differs from Rz1 by exhibiting a 4 nt shorter 3′ arm, while Rz4 was designed with a 4 nt longer 5′ arm and 1 nt shorter 3′ arm. Rz5 was engineered to contain inosine (I) and cleave the TEV 5′UTR after an ACA triplet [21]. To increase the stability of the annealed Rz:target complex, 2′-O-methylated nucleotides (Nm) were incorporated into the last 7 or 19 nucleotide positions of Rz2 and Rz5, respectively. The RNase H probe (5′-dGdAdC dCdAdG AmAmGm AmAmUm AmCmUm Am-3′), in which deoxynucleotides (dNs) were incorporated, was designed according to Beverly et al. [13]. Ribozymes and RNase H probe were synthesized by Metabion and their quality was confirmed by electrospray ionization time-of-flight mass spectrometry (ESI-TOF).

### 2.4. The mRNA Cleavage by Ribozyme

Ribozyme cleavage reactions contained 0.2 to 0.6 µM mRNA, and a 2.5-fold molar excess of ribozyme over mRNA substrate, in 10 mM Tris and 10 mM MgCl_2_. Initially, ribozyme was annealed to mRNA in the reaction mix without MgCl_2_ by incubating first at 95 °C for 2 min and then at room temperature for 5 min. The cleavage reactions were started by adding MgCl_2_ and performed at 37 °C for 1 h, then processed further or frozen at −20 °C. A 2.5-fold molar excess of ribozyme over mRNA was found to be optimal after testing a range from 1.0- to 10.0-fold molar excess of ribozyme over the mRNA substrate.

### 2.5. The mRNA Cleavage by RNase H

The RNase H cleavage assay was conducted by annealing 5-fold molar excess of RNase H probe with mRNA substrate by incubating at 92 °C for 2 min, then stepwise cooling down (65 °C for 2 min, 55 °C for 2 min, and 40 °C for 2 min) in a buffer containing 50 mM Tris and 100 mM NaCl. After annealing, 125 µM RNase H (New England Biolabs) and 10 mM MgCl_2_ were added to the reaction mix, followed by incubation at 37 °C for 1 h. The reactions were further processed or frozen at −20 °C.

### 2.6. Purification of Cleaved, Short RNA Fragments Using Silica-Based Columns

The cleaved, short RNA fragments—from a mixture of the cleaved or uncleaved long RNA fragments present in the ribozyme or RNase H-mediated cleavage reaction—were purified by adapting a procedure for RNA separation using the RNeasy Mini Kit (Qiagen, Hilden, Germany). First, 100 µL cleavage reaction mixture was mixed with 350 µL RLT buffer (lysis buffer from the RNeasy Mini Kit) and 250 µL 100% ethanol and applied to the column. The long RNAs, including the uncut, full-length mRNA and the long 3′ cleaved fragments remained on the column, while the short 5′ cleavage products were collected in the flow-through fraction. Next, 50 µL RLT buffer and 500 µL 100% ethanol were added to the collected flow-through fraction (700 µL), and the mix was applied to the second silica column sequentially in two aliquots of 625 µL with intervening centrifugation at 9600× *g* for 15 s on an Heraeus Fresco 17 Centrifuge (Thermo Fisher Scientific). Here, the temperature remained at 22 to 23 °C throughout all of the centrifugation steps. Under these conditions, the short 5′ cleavage products and ribozyme bound to the column were washed with 500 µL RPE buffer (wash buffer from the RNeasy Mini Kit) and centrifuged at 9600× *g* for 15 s, followed by a wash step with 500 µL 100% ethanol and centrifugation at 9600× *g* for 2 min. To remove the remaining ethanol, the column was transferred to a clean collection tube and centrifuged at 17,000× *g* for 1 min. Then, the column was transferred to a clean 1.5 mL tube, and the short RNA fragments were eluted by adding 30 µL RNase-free water to the column followed by centrifugation at 13,800× *g* for 1 min. Typically, 20 µg RNA was purified per column for RNAs <5 kb long or 60 µg for RNAs >5 kb long.

### 2.7. Purification of Cleaved, Short Fragments by Elution from Polyacrylamide Gel

As a second option, purification of cleaved, short fragments was performed according to a method modified from Nilsen 2013 [22], whereby separation was followed by elution from a 21% polyacrylamide gel prepared with 8 M urea. Samples were separated in two mini gels processed parallel on a Bio-Rad Mini-Protean Tetra Cell in Tris-borate-EDTA (TBE) buffer. The first gel was stained with SYBR^®^ Gold nucleic acid gel stain (S11494, Thermo Fisher Scientific) diluted 1:10,000 in TBE buffer and used as a reference, while the corresponding bands of interest were cut from the second, unstained gel run in parallel. The excised gel fragments were transferred into 400 µL elution buffer (20 mM Tris-HCl, 3 M sodium acetate, 1 mM EDTA, 0.25% SDS), frozen for 15 min on dry ice and stored overnight at room temperature to allow for the release of RNA into the solution. After centrifugation at 17,000× *g* for 10 min, the supernatant contained RNA, which was extracted with an equal volume of acid–phenol/chloroform, followed by chloroform. The isopropanol precipitation was conducted, and the recovered RNA was dissolved in 10 µL RNase-free water.

### 2.8. Visualization and Analysis of Cleaved, Short Fragments by PAGE

RNA fragments cleaved by ribozyme or RNase H were diluted 1:1 with gel loading buffer II (Thermo Fisher Scientific): (1) Directly after cleavage, (2) after cleavage and silica-column purification or (3) after purification from a gel as described above. For optimal loading, the reaction volumes were constant between the samples and the concentrations of purified short RNA fragments were between 10–70 ng/µL. RNA samples were denatured at 65 °C for 10 min, then separated using 21% PAGE, 8 M urea, in TBE buffer for 2–2.5 h, stained with SYBR^®^ Gold and visualized by ultraviolet light using a Gel Doc EZ system (Bio-Rad Laboratories, Hercules, CA, USA). The image was analyzed using Volume tools in Image Lab 5.0 software (Bio-Rad). The images of RNA bands were selected which corresponded to: (1) The slower moving capped 5′ cleavage product RNA, (2) the 1 nucleotide-shorter, faster moving uncapped 5′ cleavage product RNA, and (3) background of the same area. To quantify the capping efficiency, the relative intensities of bands corresponding to the capped and uncapped RNA were measured for each RNA sample and the background values were subtracted. The total intensity of the two bands combined was considered 100%. The calculated value for the band corresponding to the capped RNA represents the capping efficiency.

### 2.9. LC–MS Analysis of Ribozyme-Cleaved Products

The LC–MS analysis was performed using an Acquity Ultra Performance Liquid Chromatography (UPLC) system (Waters, Milford, CT, USA) coupled to a Xevo TQ-S mass spectrometer (Waters) equipped with an electrospray source operating in negative ionization mode. MS spectra were acquired over a range from *m/z* 400 to 2000. All of the samples were chromatographed on an ACQUITY UPLC® BEH C18 column (2.1 mm × 50 mm; 1.7 µm particle size, Waters) at 60 °C column temperature. The analytes were separated in a gradient of 16.6 mM triethylamine (TEA; VWR), 100 mM hexafluoroisopropanol (HFIP; Sigma-Aldrich, St Louis, MO, USA), and 10% methanol, Ultra LC–MS grade (Carl Roth, Karlsruhe, Germany) as buffer A and 16.6 mM TEA, 100 mM HFIP, and 95% methanol as buffer B, with a flow rate of 0.3 mL/min. The gradients applied for oligonucleotides >20 nt length were: 0% for 1.5 min, 0 to 7% over 3.5 min, 7 to 15% over 6.25 min, 15 to 40% buffer B over 4.5 min.

## 3. Results

### 3.1. Ribozyme Assays to Quantify the Capping Efficiency of IVT mRNA

Human alpha globin (hAg) and TEV 5′ untranslated regions (5′UTRs) are among the most widely used 5′UTRs for therapeutic IVT mRNAs [16,23,24]. Ribozymes, that targeted the 5′UTR sequences of hAg or TEV, were designed (Table 2, see Section 2). All five of the ribozymes designed here were expected to form the well-described hammerhead structure and cleave the targeted RNA after defined nucleotide triplets [19,25]. Ribozymes cleave most efficiently after the AUC or GUC triplets, while other triplets can be also targeted but with the following declining cleavage efficiency: GUA, AUA, CUC > AUU, UUC, UUA > GUU, CUA > UUU, CUU [21]. Rz1, Rz3, and Rz4 were designed to cleave after GUC and Rz2 after GUA. Rz5 contained inosine (I) which allows for recognition and cleavage after the ACA triplet in the TEV 5′UTR [21].

Each of the ribozyme-mediated cleavage reactions produced short capped and uncapped RNA 5′ cleavage products that differed from each other solely in their lengths—due to the cap structure, the capped RNA was exactly one nucleotide longer. The ribozymes were designed to cleave off 10–30 nt long products from the target RNA, while the ribozymes were 35–47 nt, allowing for the separation and differentiation of the 5′ cleavage products and the ribozyme (Table 2). The Rz2 and Rz5 sequences that are complementary to the targeted RNA also contained 2′-O-methylated nucleotides to enhance the cleavage by increasing the stabilities of the formed double-strand structures.

**Figure 1:**
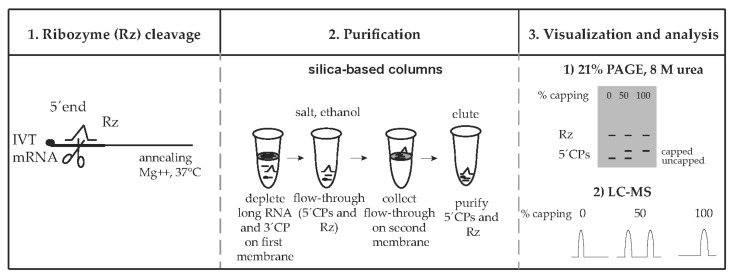
Ribozyme-mediated cleavage to quantify the capping efficiency of in vitro-transcribed mRNA. Ribozyme (Rz) anneals to IVT mRNA and cleaves the 5′-end of the IVT mRNA at 37 °C in the presence of Mg^++^. Substrate cleavage results in a mixture of RNAs: Short capped and uncapped 5′ cleavage products (5′CPs), long 3′ cleavage products (3′CPs), long uncleaved RNAs, and the Rz. The mixture is purified using a process with two silica-based columns, whereby the long RNAs and 3′CPs are depleted on the first column membrane using specific salt and ethanol conditions. The collected flow-through containing the short capped and uncapped 5′CPs is applied to the second column, bound on its membrane, and eluted in water. The purified 5′CPs and Rz are visualized using 21% PAGE, 8 M urea or analyzed with liquid chromatography and mass spectrometry (LC–MS), allowing for the quantification of capping efficiency of the IVT mRNA.

To quantify the capping efficiency, Rz was annealed to the mRNA substrate (Figure 1). In the presence of Mg^++^ ions, the 5′-end of the IVT mRNA was cleaved, resulting in a mixture of: Short capped and/or uncapped 5′ cleavage products, long 3′ cleavage products, long uncleaved RNA, and the Rz. Optimized conditions were used to purify short Rz-cleaved fragments using silica-based MinElute Qiagen columns, prior to visualization using 21% PAGE, 8 M urea or LC–MS analysis (Figure 1). In this case, 5′ cleavage products together with the ribozyme are purified and further visualized/quantified. Purification of the 5′ cleavage products is necessary for LC–MS analysis and also improves RNA visualization using 21% PAGE, 8 M urea.

An alternative purification approach was implemented on the PAGE-separated samples, by extracting and eluting the 5′ cleavage products from the gel (see Section 2, data not shown). This process results in the elimination of both the uncut and 3′-end cleaved RNA products as well as the ribozyme from the mixture, which may be an advantage in the case where the LC–MS analysis is planned using Rz and 5′ cleavage products that overlap. However, this purification step is experimentally time consuming and not easily scalable. Using this method, it is possible to purify 2–4 samples in parallel in 2 days, while using silica-based columns allows for the purification of 12 samples in parallel in less than 1 day with the option to scale up.

Here, we describe an assay to assess the capping efficiency. The method consists of a ribozyme cleavage reaction, purification of cleaved fragments, and visualization of capped and uncapped products using 21% PAGE, 8 M urea or LC–MS analysis.

### 3.2. Ribozyme Cleavage Assay Optimization

**Figure 2:**
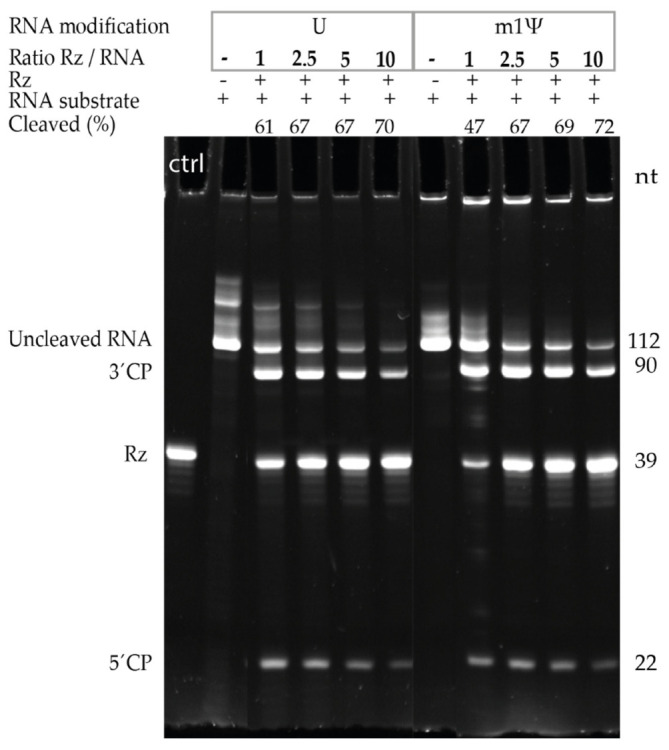
Optimization of molar ratio of ribozyme to the IVT mRNA substrate. A fixed amount of U-containing or m1Ψ-containing uncapped mRNA was cleaved using increasing amounts of Rz1, and the resulting mixture was visualized using 21% PAGE, 8 M urea. The cleavage efficiency of Rz1 was assessed for increasing Rz to IVT mRNA substrate molar ratios, based on ratios between uncleaved RNA (112 nt long) and 3′ cleavage products (3′CP = 90 nt long). Molar ratios of Rz to RNA substrate from 1 to 10 were tested, resulting in approximately 50 to 70% cleavage. Rz was used as a control (ctrl). 5′CP: 5′ cleavage products; nt: Nucleotides.

To identify the optimal molar ratio of the Rz to RNA substrate for the cleavage reaction, a 112 nt long U-containing or m1Ψ-containing mRNA substrate was selected. Using the aforementioned short substrates allowed for the detection and differentiation of the uncleaved RNA, 5′ and 3′ cleavage products, and the Rz separated on the same gel. Figure 2 shows the 112 nt long uncleaved RNA, the 22 and 90 nt long 5′ and 3′ cleavage products, and the 39 nt long Rz1 that were detected using 21% PAGE, 8 M urea. Increasing molarities of Rz over the RNA substrate were tested for both U- and m1Ψ-containing RNAs. A 2.5-fold excess molarity of Rz over the RNA substrate was selected and used in all of the subsequent experiments.

For further optimization, various temperature settings during the cleavage reaction were tested. The reaction was performed with Rz1, Rz3, and Rz4 at 25, 37, and 50 °C, as detailed in Section 2.4.

The temperature did not have any notable effect on Rz cleavage in this experiment, and the same capping efficiency results were obtained using different temperature settings. However, the reactions performed at 50 °C led to more prominent degradation (data not shown). Takagi et al. and Sawata et al. found that below 25 °C the product dissociation step has become the rate-determining step. In addition, at the temperature of 37 °C no burst kinetics were detected and ribozyme chemical cleavage was the rate-determining step [26,27]. Taking their and our findings into consideration, cleavage reactions at 37 °C were used in subsequent experiments.

### 3.3. Capping Efficiency Quantification after Visualizing 5′ Cleavage Products Using 21% PAGE, 8 M Urea

**Figure 3:**
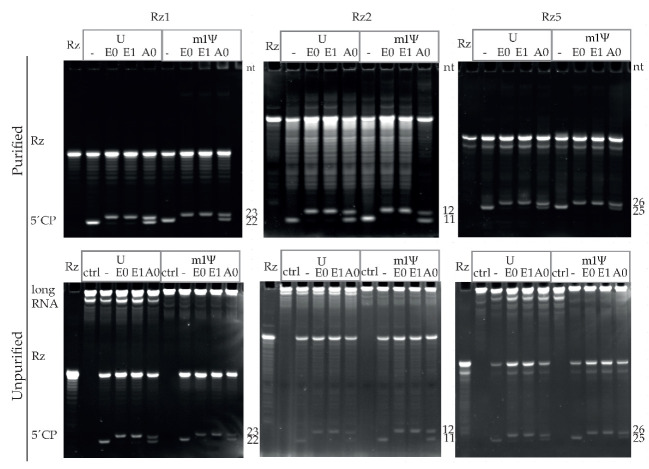
Ribozyme-mediated cleavage effectively assesses capping efficiencies of IVT mRNAs by visualization and quantification using denaturing polyacrylamide gel electrophoresis. Ribozyme- (Rz) mediated cleavage (using Rz1, Rz2, and Rz5) of U-containing or m1Ψ-containing RNAs: Uncapped (-), enzymatic cap0 (E0), enzymatic cap1 (E1), and ARCA (A0) were purified on silica-based columns and then visualized using 21% PAGE, 8 M urea (purified, upper panel) or visualized using 21% PAGE, 8 M urea without silica-based column purification (unpurified, lower panel). Rz was used as a control on both the upper and lower panels, while uncleaved mRNA was in addition used as a control (ctrl) on the lower panels. 5′CP: 5′ cleavage products (upper: Capped, lower: Uncapped); nt: Nucleotides.

To measure the capping efficiency, short capped and uncapped 5′ cleavage products were visualized using 21% PAGE, 8 M urea. As a proof-of-principle experiment, cleavage reactions were conducted using erythropoietin (EPO)-encoding mRNA. GCG transcription start site (TSS) and hAg 5′UTR-containing IVT mRNAs were cleaved using Rz1 and Rz2. EPO-encoding GGA TSS and TEV 5′UTR-containing IVT mRNAs were cleaved using Rz5. The following U- and m1Ψ-containing RNAs were tested: Uncapped (-), enzymatically capped without 2′-O-methylation (E0), enzymatically capped and 2′-O-methylated (E1) or ARCA co-transcriptionally capped (A0). Using 21% PAGE, 8 M urea, the bands representing ribozyme (Rz) and short capped and uncapped 5′ cleavage products were detected at the expected sizes (Figure 3).

The 3′-end cleavage products or uncleaved long RNAs were observed in 21% PAGE, 8 M urea gels after ribozyme-mediated cleavage reactions without a silica-based column purification step. Depletion of long RNA fragments and an increase in signals representing the short capped and uncapped 5′ cleavage products were observed after purification using silica-based columns. As expected, visualization using 21% PAGE, 8 M urea showed the presence of bands at the bottom of the gel in all of the uncapped control (-) RNA samples. Furthermore, in all of the enzymatically capped (E0 and E1) samples, a capped high-intensity band was observed in the majority of cases, while uncapped bands were not observed or had lower intensities, indicating the high capping efficiency of enzymatically capped RNAs. In contrast, both capped and uncapped bands were detected in comparable intensities in ARCA (A0) samples, irrespective of the RNA or Rz used.

Approximately 84–100% capping efficiencies were detected for all of the 12 enzymatically capped (E0 and E1) samples independent of the 5′-end and ribozyme used, while ARCA (A0) samples showed 34–53% capping efficiencies for hAg 5′UTR and 67–77% for TEV 5′UTR-containing RNAs (Table 3). When the % capping efficiencies of purified E0 RNA and corresponding 2′-O-methylated E1 RNA were compared, in five of six cases +/−1% differences were seen, showing the high reproducibility of the assay.

Capping efficiencies of silica-column purified RNAs were consistently less variable when compared to ribozyme-cleaved RNAs that were not silica-column purified. In 10 of 12 cases using different batches of purified RNAs, both E0 and E1 showed capping efficiencies between 90 and 96%, while for the same RNAs that were not silica-column purified, the observed capping efficiency range was 88 to 100% (Table 3). Therefore, purification using silica columns is recommended as a standard part of the procedure, not only for LC–MS analysis but also for visualization using 21% PAGE, 8 M urea and capping efficiency quantification.

### 3.4. Capping Assay by Ribozyme-Mediated Cleavage Effectively Assesses Capping Efficiencies of Diversely Capped IVT mRNAs of Different Lengths

**Figure 4:**
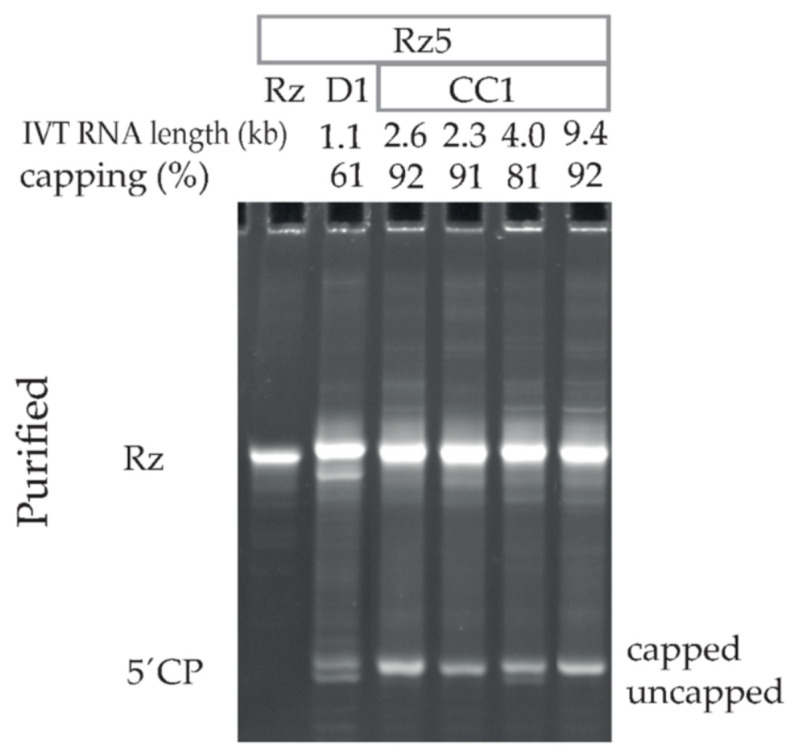
Ribozyme-mediated cleavage effectively assesses capping efficiencies of IVT mRNAs of different lengths. The image shows 21% PAGE, 8 M urea visualization of Rz5-cleaved and silica-column purified TEV, m1Ψ-containing, beta-S-ARCA (D1) or CleanCap^®^ Reagent AG (3′ OMe), cap1 (CC1) capped IVT mRNAs. The IVT mRNAs ranged from 1.1 to 9.4 kb in length. Rz: Ribozyme; 5′CP: 5′ cleavage product.

To test whether the ribozyme-mediated cleavage can detect capping of IVT mRNA with different lengths, a Rz5 cleavage reaction was performed (without modifying the method described above) on five IVT mRNAs ranging from 1.1 to 9.4 kb in length. The 1.1 kb long beta-S-ARCA (D1) capped TEV 5′UTR, m1Ψ-containing RNA showed 61% capping efficiency, while the 2.3–9.4 kb long CleanCap^®^ Reagent AG (3′ OMe), cap1 (CC1) RNAs showed 81–92% capping efficiency (Figure 4). There was no correlation observed between the % capping and RNA length. The method described here successfully assessed the capping efficiencies of IVT mRNAs of different lengths capped using diverse cap structures.

### 3.5. Ribozyme-Mediated Cleavage Assay Detects an Increase in Capping Efficiency after Additional Enzymatic Capping of Co-Transcriptionally Capped RNA

**Figure 5:**
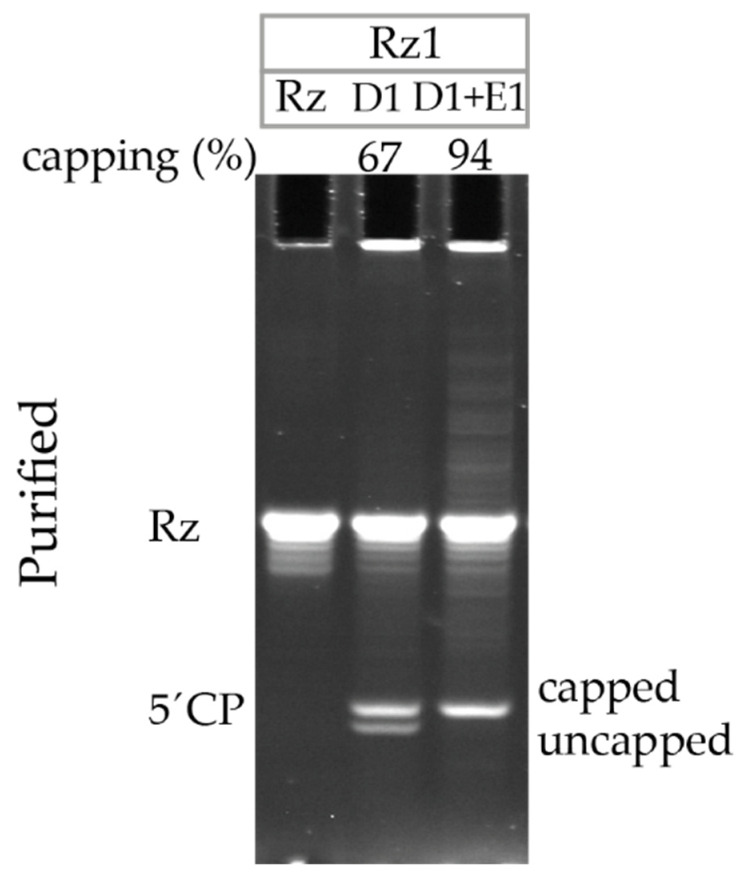
Ribozyme-mediated cleavage assay detects an increase in capping efficiency after enzymatic capping of co-transcriptionally capped IVT mRNA. GCG transcription start site (TSS), hAg, U-containing IVT mRNAs, which were co-transcriptionally D1 capped or D1 + enzymatically capped (D1 + E1), were Rz1 cleaved, silica-column purified, and visualized using 21% PAGE, 8 M urea. Rz: Ribozyme; 5′CP: 5′ cleavage product.

To further test the ribozyme-mediated cleavage assay performance, the co-transcriptionally capped D1 U-containing GCG TSS hAg 5′UTR IVT mRNA was subsequently subjected to enzymatic capping (E1). While co-transcriptionally D1 capped IVT mRNA initially showed 67% capping efficiency, after subsequent E1 capping, the capping efficiency increased to 94% (Figure 5). This finding confirms that a significant portion of 5′ cleavage products in D1 are indeed uncapped and do not represent the T7 RNA polymerase potentially skipping the first G at the start of transcription, which might result in the same RNA fragment that is 1 nt shorter than the capped fragment and equal to the size of the uncapped fragment.

### 3.6. Ribozyme-Mediated Cleavage Assay Performance Superior to RNase H Cleavage Assay

To compare the ribozyme-mediated and RNase H-mediated cleavage assays, we developed six RNase H probes that could anneal to the hAg 5′UTR sequence. RNase H cleavage reactions were performed (as described in the Section 2) and the probes were screened (data not shown). A probe (P1) containing a stretch of six DNA nucleotides (dNs) and ten 2′-O-methylated RNA nucleotides was selected due to the superior performance (Table 4).

**Figure 6:**
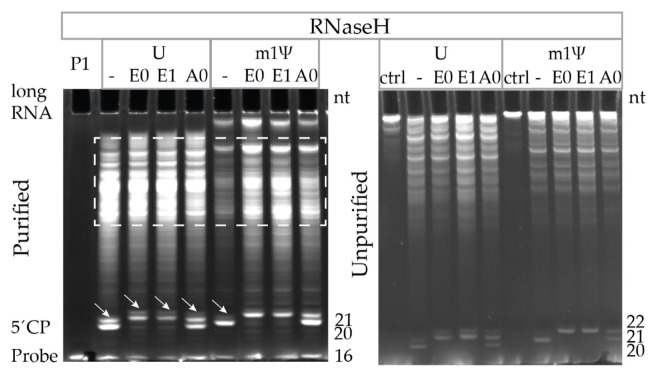
Ribozyme-mediated cleavage assay is superior to the RNase H cleavage assay. RNase H probe (P1) was hybridized, and RNase H cleaved a set of U-containing or m1Ψ-containing RNAs: Uncapped (-), enzymatic cap0 (E0), enzymatic cap1 (E1), and ARCA (A0). Cleaved RNA fragment mixtures were applied to silica-based columns for purification and visualized using 21% PAGE, 8 M urea (purified) or visualized using 21% PAGE, 8 M urea without silica-based column purification (unpurified). White arrows: Additional +1 nt band, dashed square: RNA degradation caused by RNase H. RNase H probe P1 or uncleaved RNA (ctrl) were used as controls. 5′CP: 5′ cleavage products; nt: Nucleotides.

The 5′-end of the hAg GCG-starting enzymatically capped (E0 or E1) or co-transcriptionally ARCA capped (A0) U- and m1Ψ-containing RNAs was RNase H cleaved (Figure 6). RNase H cleavage confirmed high capping efficiencies for enzymatic RNAs previously detected by ribozyme-mediated cleavage (purified hAg GCG: 75–85%). For ARCA samples, rather low capping efficiencies of 37–47% were obtained (Figure 6, Table 5), which is in accordance with the data shown in Section 3.3.

In contrast to the ribozyme-mediated cleavage, the RNase H cleavage resulted in an additional band. The band was 1 nt longer (21 or 22 nt long) and appeared in all of the U-containing samples, including uncapped RNA, next to or overlapping the expected short RNA fragments of 20 and 21 nt, corresponding to the uncapped and capped enzymatic 5′ cleavage products, respectively (Figure 6). This finding indicates that RNase H cleaved at two positions: At the expected position after the fourth DNA nucleotide and with lower efficiency at the position after the fifth DNA nucleotide, thereby complicating the capping efficiency analysis in U-containing samples (Figure 6). In addition, in all of the RNase H-cleaved samples, a large spread of nonspecific long RNA fragments not present in the no-enzyme control was observed (Figure 6). These nonspecific long RNA fragments of various sizes could not be depleted by silica-based column purification. Their presence made the capping efficiency quantification from the 21% PAGE, 8 M urea gels less reproducible, and this may lead to the complex LC–MS analysis.

Accordingly, RNase H cleavage using specific probes can be used to quantify the capping efficiency using 21% PAGE, 8 M urea. However, the ribozyme-mediated cleavage in contrast to the RNase H cleavage leads to a single-position cleavage and does not result in a smear of nonspecific long fragments, allowing for the reliable quantification using 21% PAGE, 8 M urea or LC–MS analysis.

### 3.7. LC–MS Analysis of Capping Efficiency Using the Ribozyme-Mediated Cleavage Assay

**Figure 7:**
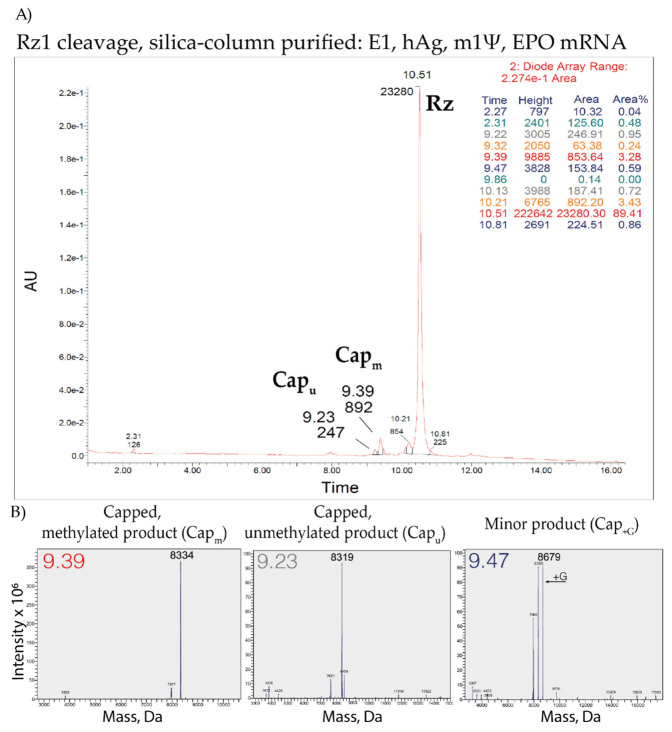
LC–MS analysis of ribozyme-mediated cleavage products for quantification and characterization of capped products from Rz1 cleaved and silica-column purified enzymatically capped and 2′-O-methylated (E1), human alpha globin (hAg), m1Ψ-containing erythropoietin (EPO) mRNA. The enzymatical capping procedure provides 7MeGpppA(OMe)GGCGAACU*AGU*AU*U*-CU*U*CU*GGU*Cp (MW = 8334) and 7MeGpppAGGCGAACU*AGU*AU*U*CU*U*CU*GGU*Cp (MW = 8319) in a 7:2 ratio, resulting from an incomplete 2′O-methyl transfer. (**A**) UPLC and (**B**) MS profiles. Rz: Ribozyme; Cap: Capped product; Cap_m_: Capped methylated product; Cap_u_: Capped unmethylated product; Cap_+G_: Minor product; AU: Arbitrary units.

As a proof-of-principle, the LC–MS analysis was conducted on ribozyme-cleaved and silica-based column purified short 5′ cleavage products from enzymatically capped and 2′-O-methylated (E1) or CleanCap^®^ Reagent AG (3′ OMe) (CC1) AGG TSS, hAg 5′UTR, m1Ψ-containing IVT mRNAs. UPLC and MS profiles of E1 capped IVT mRNA (Figure 7) showed:

- 68% of the expected major product
  (7MeGpppA(OMe)GGCGAACU*AGU*AU*U*CU*U*CU*GGU*C > p);
- 20% unmethylated product
  (7MeGpppAGGCGAACU*AGU*AU*U*CU*U*CU*GGU*C > p);
- and 1% additional product (+G).

The detection of 89% of the E1 capped product (for AGG TSS IVT mRNA) is in agreement with the detected 95–96% for E1 capped RNAs using 21% PAGE, 8 M urea. Furthermore, a different batch and GCG TSS RNA were used for PAGE.

**Figure 8:**
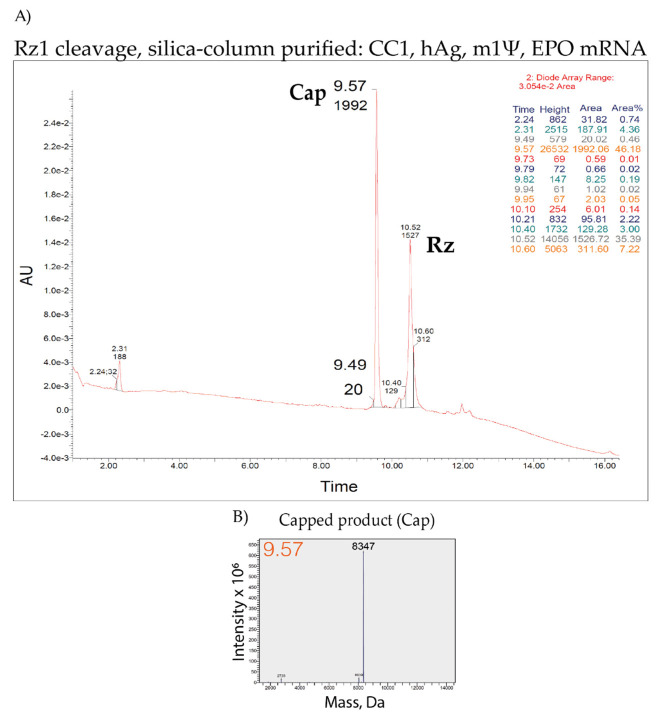
LC–MS analysis of ribozyme-mediated cleavage products for quantification and characterization of capped products from Rz1 cleaved and silica-column purified CleanCap^®^ Reagent AG (3′ OMe) = cap1 (CC1), human alpha globin (hAg), m1Ψ-containing erythropoietin (EPO) mRNA. Expected capped product (MW = 8347) is detected in >99%. (**A**) UPLC and (**B**) MS profiles. Rz: Ribozyme; Cap: Capped product; AU: Arbitrary units.

UPLC and MS profiles of CleanCap^®^ Reagent AG (3′ OMe), N-7413 TriLink (CC1) EPO mRNA (Figure 8) showed >99% capping, which is also in agreement with >90% capping efficiency obtained by screening of >100 CC1-capped IVT mRNAs using 21% PAGE, 8 M urea (Figure 4 and data not shown). Therefore, the ribozyme-mediated cleavage assay combined with silica-based column purification are highly compatible with the LC–MS analysis. As shown here, the application to LC–MS analysis creates additional possibilities for characterizing 5′ cleavage products, such as determining the methylation status or distinguishing minor capped products.

## 4. Discussion

The quality control of mRNA vaccines and therapeutics is necessary at every stage of development, from preclinical studies to clinical applications, as well as to support marketing authorizations. The cap structure on the mRNA molecule determines the mRNA translation, and thus correlates with the therapeutic efficacy of the mRNA [7,8,9]. In the process of mRNA production, capping of the mRNA can be performed enzymatically or co-transcriptionally. Both strategies result in a mixture of capped and uncapped mRNA molecules. In this study, we developed an assay to detect the capping efficiency based on a ribozyme-mediated cleavage reaction. After this reaction, the next steps are silica-column purification and visualization, and quantification using 21% PAGE, 8 M urea or LC–MS analysis. Visualization using 21% PAGE, 8 M urea allows for the analysis of a large number of mRNA samples in parallel without the need for specific equipment. In addition, the method described here is compatible with LC–MS analysis, allowing for in-depth characterization, where not only the percentage of capping, but the methylation status and potential minor capped byproducts can be detected from the same sample.

As previously discussed, the current approaches to assess the capping efficiency have limitations, such as the necessity for radioactive labeling [10,11,12] or a rather insensitive detection of capping levels [14]. Beverly et al. developed an assay to assess the capping efficiency based on the RNase H cleavage of specific biotin-tagged probes followed by purification using streptavidin-coated magnetic beads and LC–MS analysis [13]. Here, we directly compared our developed ribozyme-mediated cleavage assay, which is targeted to the hAg 5′UTR with the RNase H cleavage assay that we designed for the same 5′UTR. As expected, ribozyme cleaved only at one position in the IVT mRNA, and after silica-based purification only three short RNA fragments (the ribozyme as well as the capped and uncapped 5′ cleavage products) were observed on the gel, allowing for precise quantification. In contrast, RNase H cleaved at two positions, resulting in capped and uncapped 5′ cleavage products and an additional unexpected band 1 nt longer that also appeared in the uncapped RNA control sample. Therefore, quantification using the RNase H assay was cumbersome. These results are in accordance with the observations of Beverly et al., who also reported two cleavage sites after RNase H cleavage and biotin-tagged analysis [13]. Moreover, we observed that the RNase H cleavage also yields a large number of nonspecific long cleaved RNA fragments, which are expected to negatively influence quantification. We conclude that the assay based on the ribozyme cleavage developed in this study shows major benefits compared to the other assays for capping detection.

The ribozyme design for use in these assays depends on the 5′-terminal sequence of the target mRNA, in order to quantify the capping efficiency. Therefore, a ribozyme cleavage site is required in a structurally accessible region between position 10 and 30 nt from the 5′-end of the mRNA. In this study, hammerhead ribozymes were designed to cleave after GUC (Rz1, Rz3, Rz4), GUA (Rz2) or ACA (Rz5) triplets. In addition to these triplets, other NUH triplets (H represents A, C or U) which can be targeted by hammerhead ribozymes can be also employed (NUH cleavage efficiency in decreasing order is: AUC > AUA, CUC > AUU, UUC, UUA > GUU, CUA > UUU, CUU [21]). Incorporation of inosine in the ribozyme recognition sequence (as in Rz5) allows for additional targeting at NCH triplets (e.g., ACA). The possibility of targeting both canonical NUH triplets and non-canonical NCH sites significantly improves the versatility of this assay by allowing for the selection of the most accessible cleavage site within the 5′UTR [21]. The assays developed and optimized here could be used to reproducibly quantify the capping efficiencies of U-containing or m1Ψ-containing IVT mRNAs with diverse caps, containing different 5′UTRs, and of different lengths. The 89 to 100% capping efficiencies observed after using a vaccinia virus capping enzyme system confirm the previous findings of 88 to 98% capping, as reported by Beverly et al. [13]. Moreover, we show that diverse cap structures can lead to diverse capping efficiencies that are relatively consistent between different IVT mRNA batches produced using the same cap structure. For example, while enzymatic capping and co-transcriptionally capped CleanCap^®^ Reagent AG (3′ OMe) (TriLink) typically yielded >90% capping efficiencies, co-transcriptional capping using ARCA-G (TriLink) or beta-S-ARCA (D1) led to lower capping efficiencies ranging from 34 to 77%.

Taken together, the ribozyme-mediated cleavage assays developed in this study are useful assays for facile, fast, and reliable analysis of capping efficiency for research and development purposes or as a quality control for hAg and TEV 5′UTR-containing mRNA-based therapeutics. The same methods for ribozyme assay design, short fragment purification, and visualization or quantification by gel electrophoresis or LC–MS analysis may be used to develop ribozyme assays targeted against other 5′UTRs, allowing for a wider applicability in the mRNA therapeutics field.

## Figures and Tables

**Table 1:** Characteristics of the designed ribozymes.

5′UTR	Rz	Rz Sequence (5′-3′)
hAg	Rz1	UGU GGG CUG AUG AGG CCG UGA GGC CGA AAC CAG AAG AAU
	Rz2	GGG GAC CAG AAG AAC UGA UGA GGC CGU GAG GCC GAA ACUm AmGmUm UmCmGm
	Rz3	UGU GGG CUG AUG AGG CCG UGA GGC CGA AAC CAG AA
	Rz4	AGU CUG UGG GCU GAU GAG GCC GUG AGG CCG AAA CCA GAA GAA
TEV	Rz5	GUA UAC UGA UGA GGC CGU GAG GCC GAA IUmUm GmUmGm UmUmGm AmGmAm CmUmAm GmUmUm UmAm

Hammerhead Rz catalytic core sequences are underlined; hAg: Human alpha globin; I: Inosine; m: 2′-O-Met; TEV: Tobacco etch virus; Rz: Ribozyme; 5′UTR: 5′ untranslated region.

**Table 2:** Length of the designed ribozymes and example cleavage products.

5′UTR	Rz	Rz (nt)	Example of Uncapped 5′CP (Capped: +7MeG)	5′CP (nt)	
				−Cap	+Cap
hAg	Rz1	39	pppGCGAACUAGUAUUCUUCUGGUC > CCCACAGACU…	22	23
	Rz2	45	pppGCGAACUAGUA > UUCUUCUGGUCCCCACAGACU...	11	12
	Rz3	35	pppGCGAACUAGUAUUCUUCUGGUC > CCCACAGACU…	22	23
	Rz4	42	pppGCGAACUAGUAUUCUUCUGGUC > CCCACAGACU…	22	23
TEV	Rz5	47	pppGGAAUAAACUAGUCUCAACACAACA > UAUACAAA...	25	26

Recognition sequences are underlined; cleavage positions are indicated as N >; CP: Cleavage product; hAg: Human alpha globin; TEV: Tobacco etch virus; Rz: Ribozyme; 5′UTR: 5′ untranslated region.

**Table 3:** Capping efficiencies of IVT mRNAs quantified after ribozyme-mediated cleavage assays and visualized using 21% PAGE, 8 M urea on Figure 3.

5′UTR		hAg				TEV	
Purification		Unpurified		Purified		Unpurified	Purified
Assay		Rz1	Rz2	Rz1	Rz2	Rz5	
Cap	Modification	Capping Efficiency (%)					
E0	U	100	86	90	95	93	89
	m1Ψ	88	100	95	96	100	99
E1	U	100	84	91	95	95	90
	m1Ψ	92	100	95	96	98	95
A0	U	52	51	52	52	75	67
	m1Ψ	40	48	53	34	77	69

Enzymatically capped without 2′-O-methylation (E0); enzymatically capped and 2′-O-methylated (E1); ARCA co-transcriptionally capped (A0); unmodified/uridine-containing (U); m1Ψ-containing (m1Ψ); hAg: Human alpha globin; TEV: Tobacco etch virus.

**Table 4:** RNase H cleavage assay probe and products.

5′UTR	Probe Name	Probe Sequence (5′-3′)	Probe Size (nt)	(1) 5′CP Sequence w/o Cap	5′CP Size (nt)	
				(2) Minor Cleavage Product	−Cap	+Cap
hAg	Probe 1(P1)	**GACCAG**AmAmGmAmAmUmAmCmUmAm	16	(1) GCGAACUAGUA UUCUUCUGG	20	21
				(2) GCGAACUAGUA UUCUUCUGGU	21	22

DNA nucleotides in bold: dN; m: 2′-O-Met; hAg: Human alpha globin.

**Table 5:** Quantification of capping efficiencies of IVT mRNAs after the RNase H cleavage assay and visualization using 21% PAGE, 8 M urea.

5’UTR		hAg	
Purification		Unpurified	Purified
Cap	Modification	Capping Efficiency (%)	
E0	U	75	82
	m1Ψ	89	85
E1	U	82	78
	m1Ψ	82	83
A0	U	56	47
	m1Ψ	40	37

Enzymatically capped without 2′-O-methylation (E0); enzymatically capped and 2′-O-methylated (E1); ARCA co-transcriptionally capped (A0); unmodified/uridine-containing (U); m1Ψ-containing (m1Ψ); hAg: Human alpha globin.

## References

[1] Sahin U., Karikó K., Türeci Ö. (2014). mRNA-based therapeutics—Developing a new class of drugs. Nat. Rev. Drug Discov..

[2] Sahin U., Derhovanessian E., Miller M., Kloke B.-P., Simon P., Löwer M., Bukur V., Tadmor A.D., Luxemburger U., Schrörs B. (2017). Personalized RNA mutanome vaccines mobilize poly-specific therapeutic immunity against cancer. Nature.

[3] Pardi N., Hogan M.J., Porter F.W., Weissman D. (2018). mRNA vaccines—A new era in vaccinology. Nat. Rev. Drug Discov..

[4] Polack F.P., Thomas S.J., Kitchin N., Absalon J., Gurtman A., Lockhart S., Perez J.L., Pérez Marc G., Moreira E.D., Zerbini C. (2020). Safety and efficacy of the BNT162b2 mRNA COVID-19 vaccine. N. Engl. J. Med..

[5] Anderson E.J., Rouphael N.G., Widge A.T., Jackson L.A., Roberts P.C., Makhene M., Chappell J.D., Denison M.R., Stevens L.J., Pruijssers A.J. (2020). Safety and Immunogenicity of SARS-CoV-2 mRNA-1273 Vaccine in Older Adults. N. Engl. J. Med..

[6] Vlatkovic I. (2021). Non-Immunotherapy Application of LNP-mRNA: Maximizing Efficacy and Safety. Biomedicines.

[7] Ramanathan A., Robb G.B., Chan S.-H. (2016). mRNA capping: Biological functions and applications. Nucleic Acids Res..

[8] Shimotohno K., Kodama Y., Hashimoto J., Miura K.I. (1977). Importance of 5’-terminal blocking structure to stabilize mRNA in eukaryotic protein synthesis. Proc. Natl. Acad. Sci. USA.

[9] Paterson B.M., Rosenberg M. (1979). Efficient translation of prokaryotic mRNAs in a eukaryotic cell-free system requires addition of a cap structure. Nature.

[10] Stepinski J., Waddell C., Stolarski R., Darzynkiewicz E., Rhoads R.E. (2001). Synthesis and properties of mRNAs containing the novel "anti-reverse" cap analogs 7-methyl(3’-O-methyl)GpppG and 7-methyl (3’-deoxy)GpppG. RNA.

[11] Grudzien E., Stepinski J., Jankowska-Anyszka M., Stolarski R., Darzynkiewicz E., Rhoads R.E. (2004). Novel cap analogs for in vitro synthesis of mRNAs with high translational efficiency. RNA.

[12] Anderson B.R., Muramatsu H., Nallagatla S.R., Bevilacqua P.C., Sansing L.H., Weissman D., Karikó K. (2010). Incorporation of pseudouridine into mRNA enhances translation by diminishing PKR activation. Nucleic Acids Res..

[13] Beverly M., Dell A., Parmar P., Houghton L. (2016). Label-free analysis of mRNA capping efficiency using RNase H probes and LC-MS. Anal. Bioanal. Chem..

[14] Moya-Ramírez I., Bouton C., Kontoravdi C., Polizzi K. (2020). High resolution biosensor to test the capping level and integrity of mRNAs. Nucleic Acids Res..

[15] von Niessen A.G.O., Poleganov M.A., Rechner C., Plaschke A., Kranz L., Fesser S., Diken M., Löwer M., Vallazza B., Beissert T. (2018). Improving mRNA-Based Therapeutic Gene Delivery by Expression-Augmenting 3′ UTRs Identified by Cellular Library Screening. Mol. Ther..

[16] Karikó K., Muramatsu H., A Welsh F., Ludwig J., Kato H., Akira S., Weissman D. (2008). Incorporation of Pseudouridine Into mRNA Yields Superior Nonimmunogenic Vector With Increased Translational Capacity and Biological Stability. Mol. Ther..

[17] Kowalska J., Lewdorowicz M., Zuberek J., Bojarska E., Wojcik J., Cohen L.S., Davis R.E., Stepinski J., Stolarski R., Darzynkiewicz E. (2005). Synthesis and properties of mRNA cap analogs containing phosphorothioate moiety in 5’,5’-triphosphate chain. Nucleosides Nucleotides Nucleic Acids.

[18] Mašek T., Vopalensky V., Suchomelova P., Pospisek M. (2005). Denaturing RNA electrophoresis in TAE agarose gels. Anal. Biochem..

[19] Karikó K., Megyeri K., Xiao Q., Barnathan E.S. (1994). Lipofectin-aided cell delivery of ribozyme targeted to human urokinase receptor mRNA. FEBS Lett..

[20] Li F., Barnathan E.S., Karikó K. (1994). Rapid method for screening and cloning cDNAs generated in differential mRNA display: Application of Northern blot for affinity capturing of cDNAs. Nucleic Acids Res..

[21] Ludwig J., Blaschke M., Sproat B.S. (1998). Extending the cleavage rules for the hammerhead ribozyme: Mutating adenosine^15.1^ to inosine^15.1^ changes the cleavage site specificity from N^16.2^U^16.1^H^17^ to N^16.2^C^16.1^H^17^. Nucleic Acids Res..

[22] Nilsen T.W. (2013). Gel Purification of RNA. Cold Spring Harb. Protoc..

[23] Karikó K., Kuo A., Barnathan E.S. (1999). Overexpression of urokinase receptor in mammalian cells following administration of the in vitro transcribed encoding mRNA. Gene Ther..

[24] Holtkamp S., Kreiter S., Selmi A., Simon P., Koslowski M., Huber C., Türeci O., Sahin U. (2006). Modification of antigen-encoding RNA increases stability, translational efficacy, and T-cell stimulatory capacity of dendritic cells. Blood.

[25] Scott W.G., Horan L.H., Martick M. (2013). The Hammerhead Ribozyme: Structure, catalysis, and gene regulation. Prog. Mol. Biol. Transl. Sci..

[26] Takagi Y., Taira K. (1995). Temperature-dependent change in the rate-determining step in a reaction catalyzed by a hammerhead ribozyme. FEBS Lett..

[27] Sawata S., Shimayama T., Komiyama M., Kumar P.K.R., Nishikawa S., Taira K. (1993). Enhancement of the cleavage rates of DNA-armed hammerhead ribozymes by various divalent metal ions. Nucleic Acids Res..

